# Genomic and evolutionary characterization of Chandipura virus: a cause of the 2024 outbreak in Gujarat, India

**DOI:** 10.1128/spectrum.01578-25

**Published:** 2026-04-09

**Authors:** Nitin Shukla, Urvi Budhbhatti, Apurvasinh Puvar, Ishan Raval, Ramesh Pandit, Priyank Chavda, Anita Chauhan, Dhwani Jhala, Dhruvi Shah, Tejas Shah, Janvi Raval, Harshad Prajapati, Nilam Patel, Kamlesh Upadhyay, Madhvi Joshi, Amrutlal K. Patel, Vijay Bondre, Naveen Kumar, Chaitanya Joshi

**Affiliations:** 1 Department of Science and Technology, Gujarat Biotechnology Research Centre (GBRC), Government of Gujarat 555287 , Gandhinagar, India; 2 Health and Family Welfare Department, Government of Gujarat 163890 , Gandhinagar, India; 3 Indian Council of Medical Research (ICMR)–National Institute of Virology 29620, Pune, India; Emory University School of Medicine, Atlanta, Georgia, USA

**Keywords:** evolution, phylogenetics, AES, CHPV, Gujarat outbreak

## Abstract

Acute encephalitis syndrome (AES) caused by Chandipura virus (CHPV) is a rapidly progressive and often fatal neurological illness predominantly affecting children in India. However, limited research on CHPV disease progression and viral genomics has hindered a comprehensive understanding of its transmission dynamics and evolutionary behavior. CHPV is endemic in India, with previous outbreaks (2003–2004) reported case fatality rates (CFRs) ranging from 56% to 75%. In the current (2024) outbreak, the CFR declined to 46%, with an overall test-positivity rate of 18.6%, possibly reflecting improvements in supportive care. Despite advances in genomics and sequencing technologies, only a limited number of CHPV genomes are publicly available. To address this gap, we performed whole-genome sequencing of CHPV isolated from a pediatric patient aged 12 years from Patan, Gujarat. Comparative genomic analysis with previously reported Indian strain revealed approximately 293 mutations, including 24 non-synonymous. The estimated evolutionary rate of CHPV was ~1.62 × 10⁻² substitutions/site/year. Furthermore, the selective pressure analysis showed that, despite the virus being under strong purifying (negative) selection, several non-synonymous changes were identified. Nonetheless, as the present analysis is based on the single genome, further sequencing, validation, and broader comparative analysis are required to draw a definitive inference. However, these findings suggest that even under purifying selection pressure, CHPV retains the ability to infect and cause severe disease in children. This highlights the continued need to investigate virus–host interactions, particularly host immune responses, to better understand CHPV pathogenesis and its ability to cause disease in children.

**IMPORTANCE:**

Chandipura virus (CHPV) is an etiological agent of acute encephalitis syndrome (AES) in children, characterized by rapid neurological decline; yet the viral and host factors governing its neuropathogenesis and sudden outbreak dynamics remain poorly defined. Despite minimal genomic variation indicative of strong purifying selection, which supports the continued efficacy of existing molecular diagnostics and candidate therapeutics, CHPV re‐emerges unpredictably in human populations, as exemplified by the 2024 AES cluster in Gujarat. This outbreak underscores the importance of continuous genomic surveillance to elucidate viral behavior and immune‐evasion mechanisms. Moreover, it highlights the utility of both amplicon-based and metagenomic next‐generation sequencing approaches for future CHPV detection and comprehensive genome characterization.

## OBSERVATION

Chandipura virus (CHPV), a member of the Rhabdoviridae family and closely related to the rabies virus, has been a significant cause of an outbreak of acute encephalitis syndrome (AES) in India, especially during the monsoon season (1). *Phlebotomus* spp. were initially identified as CHPV vectors in India; however, *Sergentomyia* spp. were also later identified after CHPV RNA was isolated from them during outbreaks in Vidharbha, Maharashtra, despite their limited human contact (2). Children under the age of 15 are more vulnerable to the infection compared to adults (3). Early symptoms like fever, body aches, and headaches can quickly progress to severe neurological problems, including seizures, encephalitis, and coma, often resulting in death (4).

**Fig 1:**
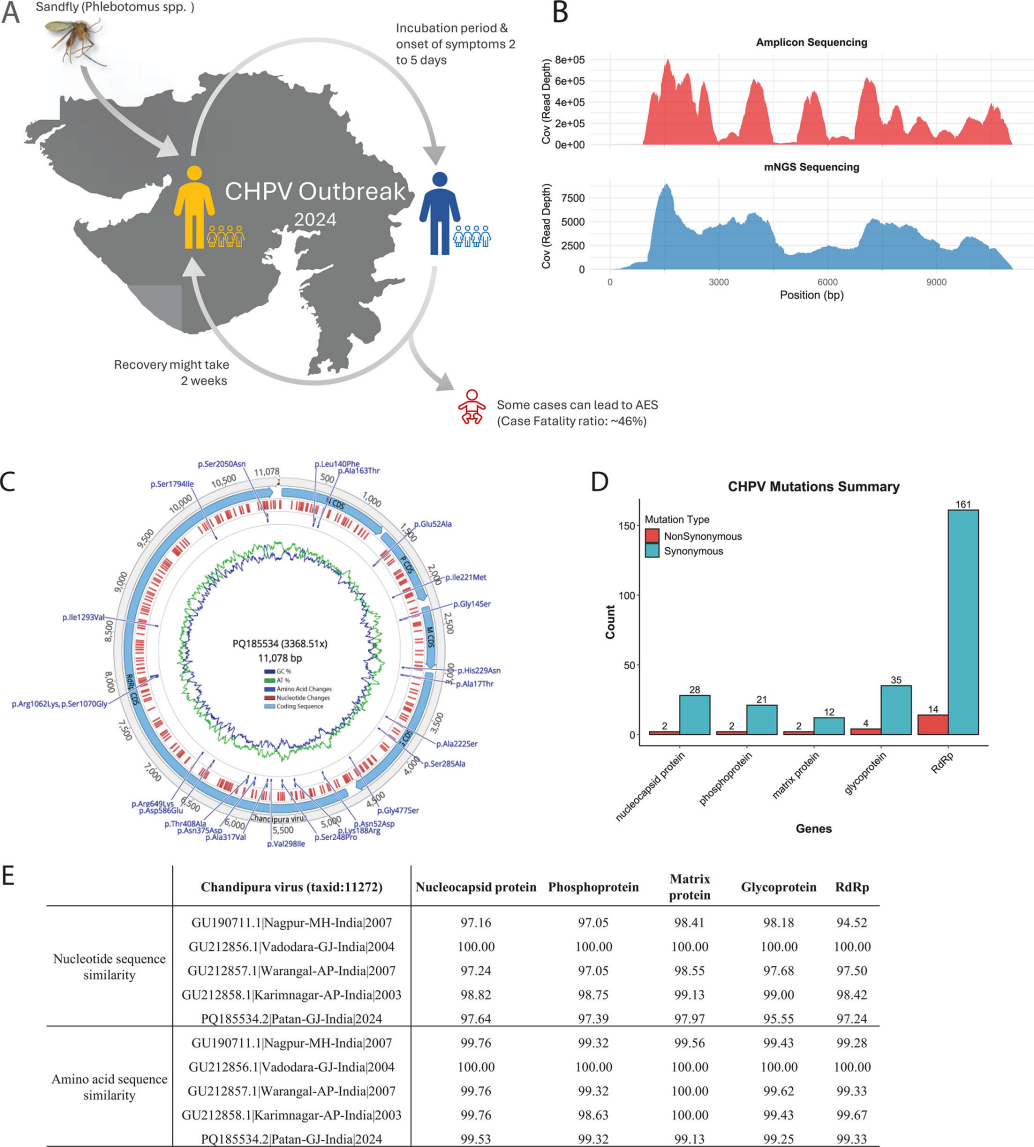
Overview of the study. (**A**) CHPV outbreak 2024, in Gujarat with a case fatality ratio of 46% among young children. The map was generated using the R packages rnaturalearth (v1.0.1) and ggplot2 (v4.0.1). (**B**) Coverage plot for CHPV genome obtained from both sequencing platforms. (**C**) The circular genome of annotated CHPV (PQ185534.2). Graph annotation from inside to outside. The blue line represents GC content, and the green line depicts the AT content of the genome. The non-synonymous amino acid changes are represented as dark blue vertical lines above, and red horizontal lines represent nucleotide changes. The genes are depicted in the form of five different segments from (nucleocapsid, phosphoprotein, matrix protein, glycoprotein, and RdRp protein). (**D**) The mutation profile of CHPV synonymous and non-synonymous changes. (**E**) Sequence similarity between the CHPV genomes against RefSeq (NC_020805.1|India|2004) at CDS and amino acid levels.

The outbreak that occurred in central India from 2003 to 2004 attracted worldwide attention, with 322 reported child fatalities: 183 in Andhra Pradesh, 115 in Maharashtra, and 24 in Gujarat. The case fatality rate (CFR) ranged from 56% to 75%, with the majority of deaths occurring within 24 hours of symptom onset (2). In 2024, Gujarat witnessed its largest CHPV outbreak in two decades, with 61 documented cases and 28 deaths by August, resulting in a cumulative CFR of ~46% as per state health records (Fig. 1A). Although significant progress has been made in understanding CHPV infections, the mechanism by which the virus invades the central nervous system (CNS) and the role of host factors in its pathogenesis still remain unclear (5). Moreover, the lack of antiviral treatments or vaccines against CHPV highlights an ongoing and serious public health concern.

Between July and December 2024, a sudden rise in AES cases was reported across multiple districts of Gujarat (Kheda, Sabarkantha, Banaskantha, Gandhinagar, Mehsana, and Godhra). The Gujarat Biotechnology Research Center was designated as a nodal center for CHPV testing by the Health and Family Welfare Department, Government of Gujarat. A total of 328 AES-suspected pediatric cases (1–16 years) were screened, of which 61 were CHPV positive (18.59%), which included 35 males and 26 females. One fatal index case, a 12-year-old male from Patan (Lab ID: CHPV0744), presented with high-grade fever, vomiting, and convulsions, with symptom onset on 18 July 2024. He was hospitalized at GMC Patan, and clinical samples (CSF, plasma, serum, swab, and urine) were collected on 20 July 2024. Despite intensive care, the patient succumbed to the illness, and the CHPV infection was confirmed by RT-PCR, predominantly in serum, prompting genomic characterization of the strain.

Two methods were adopted to obtain the genome of the virus. First, a custom amplicon panel of 17 overlapping amplicons (30 primers and additional 2 primers) (Fig. S1 and Table S1) was designed for targeted sequencing on the Ion GeneStudio S5 Plus System, which generated ~21 million QC filtered reads (~3 Gb), of which ~76% mapped to NC_020805.1, providing a coverage of ~0.2 million. Second, metagenomic next-generation sequencing (mNGS) assay performed in Illumina MiSeq yielded ~0.5 million reads (237.17 Mb); after de-hosting, ~0.1 million reads mapped to CHPV. Illumina-based mNGS provided improved terminal coverage and identified three additional nucleotide substitutions and 45 extra bases at the 5′ end (Fig. 1B and Table S2). The final consensus genome, resolved using mNGS, was submitted to GenBank (accession number PQ185534.2) (Fig. 1C). The reconstructed genome is 11,079 bp, with 30.70% A, 26.61% T, 23.13% G, and 19.55% C (GC content 42.7%), and shared 97.36% nucleotide similarity with the RefSeq genome. In total, 293 single-nucleotide variations (SNVs) were identified relative to the reference (NC_020805.1), including 12 intergenic and 257 synonymous substitutions. The protein-wise distribution includes glycoprotein (G, 35 SNVs), nucleocapsid (N, 28), phosphoprotein (P, 21), matrix protein (M, 12), and RNA-dependent RNA polymerase (RdRp, 161). Twenty-four were missense mutations: N (L140F and A163T), G (A17T, A222S, S285A, and G477S), RdRp (N52D, K188R, S248P, V298I, A317V, N375D, T408A, D586E, R649K, R1062K, S1070G, I1293V, S1794I, and S2050N), P (E52A and I221M), and M (G14S and H229N). Based on side-chain properties (functional R groups), most amino acid changes were neutral (Fig. 1D and E; Table S3).

**Fig 2:**
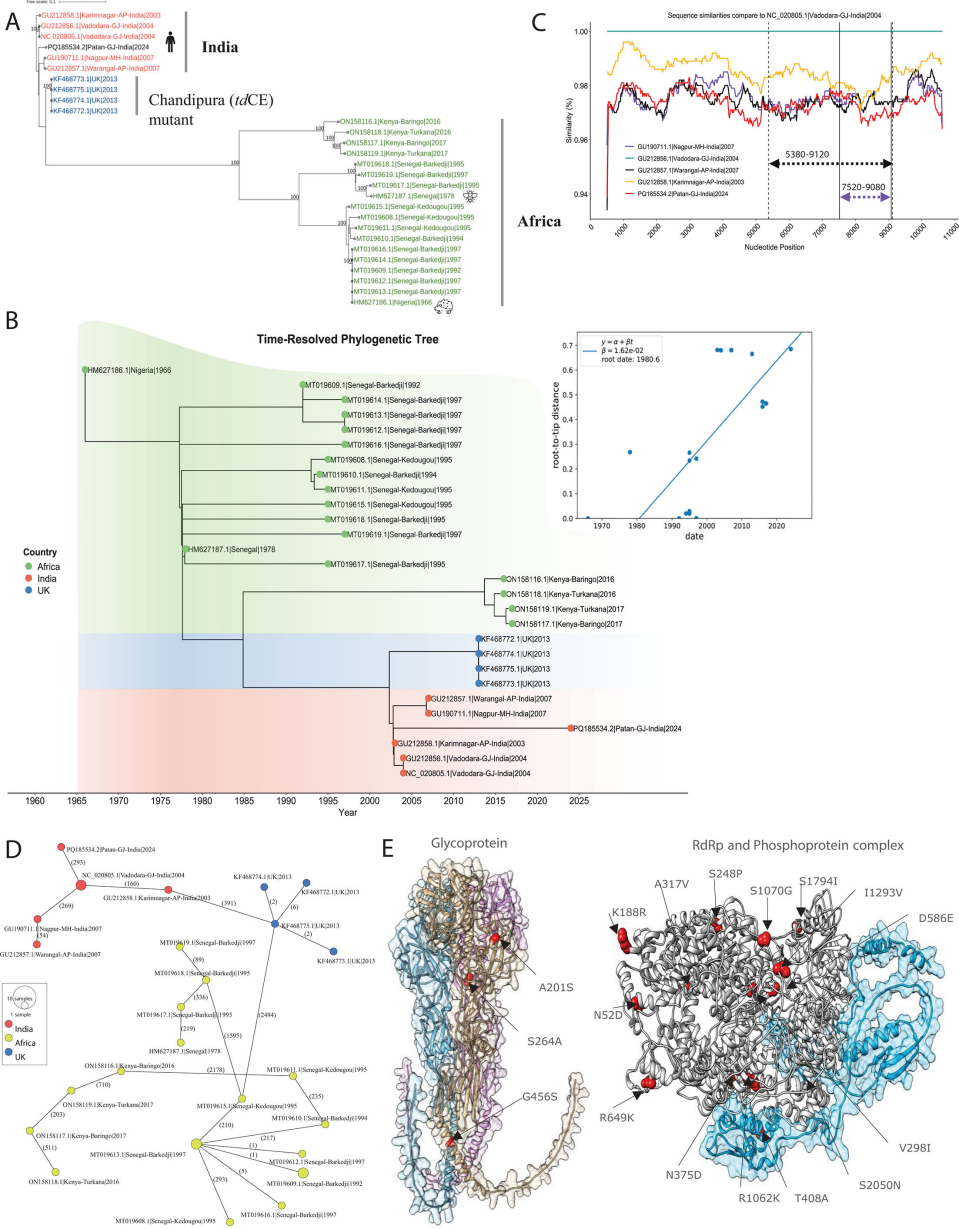
Phylodynamics and mutational highlights of the CHPV virus. (**A**) Phylogenetic tree of CHPV genomes. A total of 28 sequences from the NCBI Virus were analyzed, and the date of sample collection ranged from 1966 to 2024. The sequences highlighted in red are submitted from India, and the black color with accession number PQ185534.2 is from the current study. The sequences highlighted in blue were of the CHPV (*td*CE) mutant submitted by the University of Warwick (UK), and sequences that are highlighted in green were reported from Africa. (**B**) Time-resolved CHPV phylogenetic tree. Time tree from the earliest date to the very recent CHPV genome. Note that sequences labeled as from the UK were not originally isolated in the UK but represent *td*CE mutants generated *in vitro*. Root-to-tip estimate regression analysis. (**C**) Sequence similarity and recombination between CHPV genomes from India. (**D**) The network uses a minimum spanning method, where each node represents a virus. Each haplotype is shown as a circle, with the size indicating a common haplotype. The lines between nodes represent mutational steps, and the length of the line shows the number of mutations. The network illustrates the genetic relationship and distance between the 28 genomes, with node size reflecting haplotype frequency. (**E**) Structural representation of the mutations in glycoprotein and RNA-dependent RNA polymerase (RdRp).

Phylogenetic analysis of global CHPV genomes delineated three clades: Clade I (Indian strains, including PQ185534.2), Clade II (*td*CE mutant lineage), and Clade III (African strains). The Gujarat 2024 genome clustered within Clade I with minimal divergence from previously reported CHPV genomes from India (2003–2007), indicating that the recent CHPV genome is an endemic lineage rather than emergence of a novel variant (Fig. 2A). Time-resolved phylogeny estimated an evolutionary rate of 1.62 × 10^−^² substitutions/site/year or approximately 1.62 substitutions per 100 sites annually with (*r*² = 0.49), a moderate fit between time and mutations (Fig. 2B).

Simplot++ and Proportion test based on the Kimura 2-parameter distance model, detected strong recombination signals between PQ185534.2|Patan-GJ-India|2024 and GU190711.1|Nagpur-MH-India|2007 in the region spanning positions 7,520–9,080 (score: 74.69) and between PQ185534.2|Patan-GJ-India|2024 and GU212857.1|Warangal-AP-India|2007 in the region spanning positions 5,380–9,120 (score: 72.53) (Fig. 2C). However, given the geographically restricted outbreak and limited evidence, recombination is unlikely to be the primary driver of the 2024 event. These findings highlight the need for continued genomic and vector surveillance to clarify the contribution of recombination to CHPV evolution.

The haplotype network of CHPV genomes showed three distinct clusters corresponding to Indian strains, African strains, and the CHPV (*td*CE) mutant, reflecting clear regional genetic signatures. Nodes represented unique haplotypes, with larger nodes indicating groups of similar sequences. The 2024 outbreak strain PQ185534.2|Patan-GJ-India|2024 formed a distinct node but remained closely linked to NC_020805.1|India|2004 and other Indian strains, consistent with a shared evolutionary origin and recent diversification. In contrast, African strains and the *td*CE mutant formed genetically divergent clusters, demonstrating significant genetic divergence from the sequences submitted from India, highlighting distinct evolutionary trajectories, region-specific genetic signatures, and potential localized evolutionary pressures (Fig. 2D).

The selection pressure dN/dS ratios for RdRp (0.049), G (0.05), M (0.05), P (0.06), and N (0.01) were all <1, indicating strong purifying (negative) selection on structural and replication proteins, which suggests that these genes are highly conserved throughout the viral evolution, playing a key role in viral replication and pathogenicity essential for virus fitness and survival over time (Table S4). Despite minimal non-synonymous changes, (Fig. 2E) CHPV remains capable of infecting children and causing significant harm (6), suggesting that the virus prioritizes genomic integrity. This may be attributed to its short replication cycle in the host (7), which limits the opportunity for accumulating mutations. Furthermore, *in silico* protein stability predictions (I-Mutant, INPS, and MutPred2) highlighted two substitutions of particular interest: L140F in N, predicted to destabilize protein structure, and S1794I in RdRp, predicted to increase stability. Among G protein changes, A222S lies within a discontinuous antigenic site spanning residues (53–63, 215–229, and 260–273) but appears to be under neutral selection with no predicted major impact on stability. These observations suggest that, despite mutations at functionally relevant loci, CHPV largely maintains structural and functional integrity.

In conclusion, this study elucidates the genome of a pediatric fatal case of CHPV during the 2024 outbreak in Gujarat. The virus responsible for current infections in children shows some amino acid changes compared to the 2003–2007 isolates. However, overall, the virus exhibits few non-synonymous mutations, indicative of negative (purifying) selection. Consequently, the observed fatalities may primarily result from the host’s immune response to the virus. Understanding host–virus interactions, particularly those leading to neurological complications and AES, is vital for addressing the pathogenicity of the virus and its ability to reach the CNS. However, as this study is based on the single isolate, further comparative analysis and focused studies are required to gain deeper insights into the virus’s behavior and its impact on public health.

## Data Availability

The final genome sequence generated in this study has been deposited in the GenBank database of the National Center for Biotechnology Information under accession number PQ185534.2.

## SUPPLEMENTAL MATERIAL

Figure S1 spectrum.01578-25-s0001.eps Amplicon sequencing method.

Supplemental material spectrum.01578-25-s0002.docx Supplemental methods, legend for Fig. S1, and Tables S1 to S4.

ASM does not own the copyrights to Supplemental Material that may be linked to, or accessed through, an article. The authors have granted ASM a non-exclusive, world-wide license to publish the Supplemental Material files. Please contact the corresponding author directly for reuse.
