# Supplementary material for: Genomic and evolutionary characterization of Chandipura virus: a cause of the 2024 outbreak in Gujarat, India
Source: Microbiol Spectr. 2026 Apr 9;14(5):e01578-25. doi: 10.1128/spectrum.01578-25 (PMC13141928; doi:10.1128/spectrum.01578-25)
Supplement: Supplemental material — Supplemental methods, legend for Fig. S1, and Tables S1 to S4. [file spectrum.01578-25-s0002.docx]

**Supplementary**

**RNA Extraction and qRT-PCR**

Viral RNA was extracted using the QIAamp Viral RNA Mini Kit (Qiagen, Cat# 52906) per the manufacturer’s instructions. Viral load was quantified using the Quantiplus Chandipura Virus Detection Kit (Huwel Lifesciences, India). Two different enrichment strategies were employed for viral genome recovery: a targeted amplicon-based approach using custom-designed primers for Chandipura virus, and an untargeted metagenomic next-generation sequencing (mNGS) assay to capture broader viral content directly from patient serum.

**Amplicon Design, PCR and Sequencing**

A custom amplicon was designed, using available CHPV genome sequences submitted from

India (taxid: 11272) which included following accessions: GU212858.1, GU212856.1, NC_020805.1, GU190711.1, and GU212857.1. The multiple sequence alignment of the sequences was carried out using MAFFT (v7.520) (1). The aligned fasta was subjected to varVAMP tiled amplicon primer design (optimum length: 800, minimum overlap: 100, maximum length:1000) (v.1.2.0) which designed primers and overlapping amplicons for complete virus genome (Supplementary Figure 1).

The cDNA was prepared from the RNA isolated from patient serum using Ion Torrent NGS Reverse Transcription Kit (Cat A45003) as per manufacturer’s protocol. For amplicon PCR, separate reactions were kept with Pool1 and Pool2 custom primers using KAPA HiFi HotStart

ReadyMix (Roche, Switzerland) with cycling conditions of initial denaturation at (95°C, 2 min), followed by 30 cycles of denaturation (98°C, 30 sec), annealing (60°C, 30 sec), extension (72°C, 1 min), and a final extension at (72°C, 5 min). PCR products were resolved with a 1.2%

agarose gel, and bands were purified using gel extraction kit. DNA was quantified using a Qubit 1X dsDNA High Sensitivity (HS) kit using Qubit Fluorometer v4.0 (Thermo Fisher Scientific, MA, USA). The amplified product was purified using 0.8X QIAseq beads (Qiagen, Germany). Equal amounts of DNA from both the pools were used for shotgun library preparation with Ion Plus Fragment Library Kit (Thermo Fisher Scientific, MA, USA). The sequencing was carried out on the Ion GeneStudio™ S5 Plus System with 530 chip and 400 bp chemistry.

**Amplicon Data Analysis**

The raw FASTQ reads underwent quality filtering using Trimmomatic (v.0.39) (2). Following this, the high-quality trimmed reads were aligned to the reference genome (NC_020805.1) using minimap2 (v.2.26-r1175) (3), and the aligned data was processed using ViralConsensus (v.0.0.5) (4) to generate the consensus genome sequence of the virus from mapped reads along with primer bed file. Subsequently, bcftools (v.1.14) (5) was used for the variant calling, and the consensus genome was analyzed for mutations to identify changes in nucleotides relative to the reference genome.

**mNGS Assay and ILLUMINA Shotgun Sequencing**

For the double-stranded cDNA synthesis, the primer annealing was performed using a set of

96 Endoh primers (6) at 95°C for 5 minutes and ramped down to 20°C. It was placed on ice immediately and processed for first strand synthesis using ProtoScript II First Strand cDNA synthesis kit (Cat no. E6560, New England Biolabs, Germany). The reaction mixture was incubated at 25°C for 5 minutes, 42°C for 1 hour, 80°C for 5 minutes and hold at 4°C. For the second strand cDNA synthesis, NEBNext Ultra II (non-directional) RNA second strand synthesis kit (Cat no. E6111L, New England Biolabs, Germany) were used. The reaction mixture was incubated at 16°C for 1 hour. After being purified with (1.2X) DNA purification beads, the generated cDNA was eluted in 25μl nuclease free water. The cDNA was then amplified using the FR20RV primer (GCCGGAGCTCTGCAGATATC) using KAPA HiFi HotStart ReadyMix with cycling conditions of initial denaturation at (95°C, 2 min), followed by 30 cycles of denaturation (95°C, 10 sec), annealing (60°C, 15 sec), extension (72°C, 1 min), and a final extension at (72°C, 5 min). The PCR amplified DNA was processed for shotgun library preparation using Illumina Nextera XT Library preparation kit with 5 minutes tagmentation. The library was quantified using Qubit 1X dsDNA HS on Qubit Fluorometer v4.0. The sequencing was performed on Illumina MiSeq using v2 500 cycles (250 × 2 chemistry).

**mNGS Data Analysis**

The quality of the sequencing data was assessed using FASTQC (v0.12.1) (7). Primer sequences were removed from the raw FASTQ dataset using fastp (v0.24.0) (8). Host-derived sequences were filtered by mapping the dataset to the T2T-CHM13v2.0 assembly of the human genome (GCF_009914755.1) with Bowtie2 (v2.5.1) (9). For reference-based alignment, unmapped reads were aligned to the RefSeq sequence of CHPV (NC_020805.1) using minimap2 (v2.26-r1175) (3). A consensus sequence was generated using samtools mpileup (v1.21) (5), combined with iVar consensus (v1.4.3) (10). Finally, the variants were identified with iVar tool. The sequence was annotated using VADR (v.1.6.4) (11) and deposited in GenBank under the accession (PQ185534.2).

**Phylogenetic and Recombination**

The genome sequences of CHPV were downloaded from the NCBI virus (n=28) along with metadata; only those sequences were considered in the analysis that had a length greater than 11,000 nt, and the alignment of the sequences was performed using MAFFT (v.7.525) (1). Phylogenetic analysis of the aligned sequences was done with RAxML (v.8.2.12) (12), with 1000 bootstraps and a General Time Reversible (GTR) model with Gamma distribution for rate heterogeneity. The phylogenetic tree was visualized in ITOL (<https://itol.embl.de>) (13). We also investigated time-scale phylogeny and root-to-tip (RtT) regression analysis with TreeTime (v.0.11.4) (14). The recombination events were assessed among the sequences available from India, using the aligned fasta sequence and similarity plot generated with SimPlot++ (v.1.3) (window size:1000 bp; step:20bp) (15,16). Lastly, the phylogeographic pattern of CHPV was explored by conducting a haplotype network analysis using PopART (v.1.7) with minimum spanning network and epsilon set to zero (16,17).

**Selection Pressure Analysis**

Selection pressure across multiple genes was assessed using aligned CDS sequences, with the ratio of nonsynonymous to synonymous substitutions (dN/dS) estimated via the Datamonkey web server ([www.datamonkey.org](http://www.datamonkey.org)) (18,19). Three different methods were applied to the aligned CDS sequences: SLAC (Single-Likelihood Ancestor Counting), FUBAR (Fast, Unconstrained Bayesian AppRoximation for Inferring Selection), and FEL (Fixed Effects Likelihood). These methods categorize selection as neutral (dN/dS = 1), positive (dN/dS > 1), or negative/purifying (dN/dS < 1).

**Impact of Mutation on Protein Stability**

Protein stability is the important parameter that regulates its activity and function. Predicting the effect of the mutation on the conformational and structural integrity of the protein can shed light on the functional impact of the mutation. For that, we have analyzed the alteration in the free energy change of protein folding using I-Mutant (v2.0) (20), MutPred2 (21), and INPS (22,23) webtools. The wild-type structures of the Glycoprotein and RdRp proteins were predicted using the AlphaFold server (24). The resulting CIF files were subsequently imported into Chimera (25) for mutation annotation.

**Figure S1** The amplicon sequencing method: A) The representative multiple sequence alignment of five sequences of CHPV available from India. B) Overlapping amplicon plot representing 15 amplicons in blue color, 16 primers in pool 0 and 14 primers in pool 1 highlighted in red color.

**Supplementary Table 1.** Custom amplicon primer sequences designed for complete Chandipura virus genome amplification.

| **Amplicon Name** | **Sequence** | **Start** | **Stop** |
| --- | --- | --- | --- |
| CHA1F1 | ACGAAGACAAAAAAACCATTTAAACG | 1 | 26 |
| CHA1F | TTCTGCATTTCTACAGGTCAAACA | 46 | 69 |
| CHA1R | CGATGTCRACTGGRACTAACGC | 979 | 1000 |
| CHA2F | ACCCAAAGCTTCAGGACACTCT | 1365 | 1386 |
| CHA2R | ATCGCCCTTTTCTCTCTTGGCT | 2303 | 2324 |
| CHA3F | AACCACTCAAGGACGGGCATTA | 2687 | 2708 |
| CHA3R | GCCTGCAGCRATTTCTTTGGT | 3660 | 3680 |
| CHA4F | ATGCTCAGTCTATTGCCGACGA | 4327 | 4348 |
| CHA4R | ACTCATTATCGGTRGCGGCAT | 5227 | 5247 |
| CHA5F | TATTCGGAAGCGTTGGCCAGT | 5853 | 5873 |
| CHA5R | TTGATTGTCGCCTTGKGCTAGC | 6824 | 6845 |
| CHA6F | AGTGTGTGGTACRGCCCTTTCT | 7355 | 7376 |
| CHA6R | TTGCTTTCCTTCTCCCAAGGCT | 8191 | 8212 |
| CHA7F | GCAGACGACAGTTCAATCTTCCC | 8838 | 8860 |
| CHA7R | TAACGTAGRACTGCYGCTGTCA | 9721 | 9742 |
| CHA8F | CGCTCCTCACCCAGATTGGATAT | 10232 | 10254 |
| CHA8R | TGTACACAACATGGTGCTTTATCA | 11028 | 11051 |
| CHA9F | TGATCGACATGGGCTTGTCCA | 866 | 886 |
| CHA9R | AGTCTCTCCGCCAATGCATGA | 1744 | 1764 |
| CHA10F | AAACCGAATCACCTGGCTCCA | 1917 | 1937 |
| CHA10R | TTGCCGCCCCTTCAATCATTTC | 2886 | 2907 |
| CHA11F | TTGTTGGAGGAGAATGCGACCA | 3523 | 3544 |
| CHA11R | AAACAACACCGGCAGCTAAGC | 4435 | 4455 |
| CHA12F | TAAGGGGRTGGACTGGTGGAGA | 5110 | 5131 |
| CHA12R | TGGCCATGTGTTGTCTCGGAT | 5973 | 5993 |
| CHA13F | AGAGGGTTTGCTGGAATGGACA | 6703 | 6724 |
| CHA13R | TGCAGCACTCATTCCCATCGA | 7563 | 7583 |
| CHA14F | ARTGTTGCAAGGAGTCYCGAA | 8059 | 8079 |
| CHA14R | GCAACACTTCKCCTGTGGATAACC | 8942 | 8965 |
| CHA15F | CCAAAGAAYTTGAAGCCCTGTCC | 9558 | 9580 |
| CHA15R | TTGCAGCRGTGATCTGAGTCA | 10468 | 10488 |
| CHA15R1 | TACGAAGAAAACAAAACCAG | 11101 | 11120 |

**Supplementary Table 2.** Comparative between amplicon and mNGS assay

| **Sample ID** | **Method** | **Sequencing**  **Technology** | **Assembly Method** | **Coverage** | **Consensus length** |
| --- | --- | --- | --- | --- | --- |
| CHPV0744 | Amplicon | Ion GeneStudio™ S5 Plus System | viral_consensus (v.0.0.5) | 251541.26x | 11037 bp |
|  | mNGS | MiSeq Illumina | iVar (v.1.4.3) | 3368.51x | 11078 bp |

**Supplementary Table 3.** Nonsynonymous mutations identified in 5 proteins of CHPV isolate (PQ185534.2).


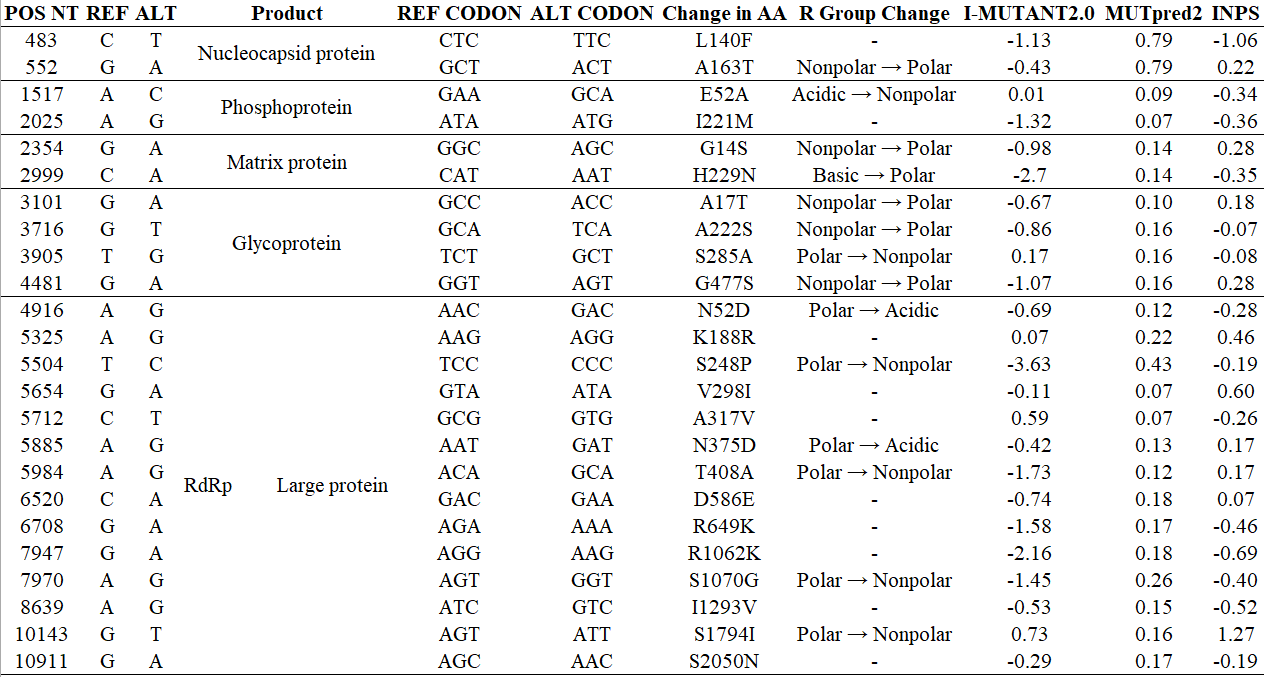


**Supplementary Table 4.** Summary of selection pressure analysis methods applied to CHPV genes: SLAC (ML and counting, P ≤ 0.1), FUBAR (Bayesian approach posterior probability, P ≥ 0.9), and FEL (ML-based, P ≤ 0.1). These methods identify sites under purifying/negative or positive selection.

| **Gene** | **Method** | **Number of positive Selection Sites P [dN/dS > 1]** | **Number of Negative Selection Sites P [dN/dS < 1]** | **dN/dS** |
| --- | --- | --- | --- | --- |
| RdRp | SLAC | 0 | 284 (13.58%) | 0.049 |
|  | FUBAR | 0 | 280 (13.38%) | - |
|  | FEL | 0 | 152 (7.27%) | - |
| Glycoprotein | SLAC | 0 | 62 (11.70%) | 0.05 |
|  | FUBAR | 0 | 51 (9.62%) | - |
|  | FEL | 0 | 31 (5.85%) | - |
| Matrixprotein | SLAC | 0 | 21 (9.17%) | 0.05 |
|  | FUBAR | 0 | 17 (7.42) | - |
|  | FEL | 0 | 7 (3.06%) | - |
| Phosphoprotein | SLAC | 0 | 41 (13.99%) | 0.06 |
|  | FUBAR | 1 | 26 (8.87%) | - |
|  | FEL | 0 | 22 (7.51%) | - |
| Nucleocapsid | SLAC | 0 | 56 (13.27%) | 0.01 |
|  | FUBAR | 0 | 51 (12.09) | - |
|  | FEL | 0 | 23 (5.45%) | - |

**References**

1. Nakamura T, Yamada KD, Tomii K, Katoh K. Parallelization of MAFFT for large-scale multiple sequence alignments. Bioinformatics [Internet]. 2018;34(14):2490–2. Available from: https://doi.org/10.1093/bioinformatics/bty121

2. Bolger AM, Lohse M, Usadel B. Trimmomatic: a flexible trimmer for Illumina sequence data. Bioinformatics [Internet]. 2014;30(15):2114–20. Available from: https://doi.org/10.1093/bioinformatics/btu170

3. Li H. Minimap2: pairwise alignment for nucleotide sequences. Bioinformatics [Internet]. 2018;34(18):3094–100. Available from: https://doi.org/10.1093/bioinformatics/bty191

4. Moshiri N. ViralConsensus: a fast and memory-efficient tool for calling viral consensus genome sequences directly from read alignment data. Bioinformatics [Internet]. 2023;39(5):btad317. Available from: https://doi.org/10.1093/bioinformatics/btad317

5. Danecek P, Bonfield JK, Liddle J, Marshall J, Ohan V, Pollard MO, et al. Twelve years of SAMtools and BCFtools. Gigascience [Internet]. 2021;10(2):giab008. Available from: https://doi.org/10.1093/gigascience/giab008

6. Endoh D, Mizutani T, Kirisawa R, Maki Y, Saito H, Kon Y, et al. Species-independent detection of RNA virus by representational difference analysis using non-ribosomal hexanucleotides for reverse transcription. Nucleic Acids Res. 2005;33(6):1–11.

7. Simon Andrews FK, Segonds-Pichon A, Biggins L, Krueger C, Wingett S. FastQC: a quality control tool for high throughput sequence data. 2010.

8. Chen S, Zhou Y, Chen Y, Gu J. Fastp: An ultra-fast all-in-one FASTQ preprocessor. Bioinformatics. 2018;34(17):i884–90.

9. Langmead B, Salzberg SL. Fast gapped-read alignment with Bowtie 2. Nat Methods. 2012;9(4):357–9.

10. Castellano S, Cestari F, Faglioni G, Tenedini E, Marino M, Artuso L, et al. Ivar, an interpretation‐oriented tool to manage the update and revision of variant annotation and classification. Genes (Basel). 2021;12(3).

11. Schäffer AA, Hatcher EL, Yankie L, Shonkwiler L, Brister JR, Karsch-Mizrachi I, et al. VADR: Validation and annotation of virus sequence submissions to GenBank. BMC Bioinformatics. 2020;21(1):1–23.

12. Stamatakis A. RAxML version 8: A tool for phylogenetic analysis and post-analysis of large phylogenies. Bioinformatics. 2014;30(9):1312–3.

13. Letunic I, Bork P. Interactive Tree of Life (iTOL) v6: Recent updates to the phylogenetic tree display and annotation tool. Nucleic Acids Res. 2024;52(W1):W78–82.

14. Sagulenko P, Puller V, Neher RA. TreeTime: Maximum-likelihood phylodynamic analysis. Virus Evol. 2018;4(1):1–9.

15. Samson S, Lord É, Makarenkov V. SimPlot++: a Python application for representing sequence similarity and detecting recombination. Bioinformatics. 2022;38(11):3118–20.

16. Sendow I, Meki IK, Dharmayanti NLPI, Hoerudin H, Ratnawati A, Settypalli TBK, et al. Molecular characterization of recombinant LSDV isolates from 2022 outbreak in Indonesia through phylogenetic networks and whole-genome SNP-based analysis. BMC Genomics. 2024;25(1):1–12.

17. Leigh JW, Bryant D. popart: full-feature software for haplotype network construction. Methods Ecol Evol [Internet]. 2015 Sep 1 [cited 2025 Jan 17];6(9):1110–6. Available from: https://onlinelibrary.wiley.com/doi/full/10.1111/2041-210X.12410

18. Weaver S, Shank SD, Spielman SJ, Li M, Muse S V., Kosakovsky Pond SL. Datamonkey 2.0: A modern web application for characterizing selective and other evolutionary processes. Mol Biol Evol. 2018;35(3):773–7.

19. Kosakovsky Pond SL, Poon AFY, Velazquez R, Weaver S, Hepler NL, Murrell B, et al. HyPhy 2.5 - A Customizable Platform for Evolutionary Hypothesis Testing Using Phylogenies. Mol Biol Evol. 2020;37(1):295–9.

20. Capriotti E, Fariselli P, Casadio R. I-Mutant2.0: Predicting stability changes upon mutation from the protein sequence or structure. Nucleic Acids Res. 2005;33(SUPPL. 2):306–10.

21. Pejaver V, Urresti J, Lugo-Martinez J, Pagel KA, Lin GN, Nam HJ, et al. Inferring the molecular and phenotypic impact of amino acid variants with MutPred2. Nat Commun [Internet]. 2020;11(1). Available from: http://dx.doi.org/10.1038/s41467-020-19669-x

22. Fariselli P, Martelli PL, Savojardo C, Casadio R. INPS: Predicting the impact of non-synonymous variations on protein stability from sequence. Bioinformatics. 2015;31(17):2816–21.

23. Savojardo C, Fariselli P, Martelli PL, Casadio R. INPS-MD: A web server to predict stability of protein variants from sequence and structure. Bioinformatics. 2016;32(16):2542–4.

24. Abramson J, Adler J, Dunger J, Evans R, Green T, Pritzel A, et al. Accurate structure prediction of biomolecular interactions with AlphaFold 3. Nature. 2024;630(8016):493–500.

25. Pettersen EF, Goddard TD, Huang CC, Couch GS, Greenblatt DM, Meng EC, et al. UCSF Chimera - A visualization system for exploratory research and analysis. J Comput Chem. 2004;25(13):1605–12.
